# How to Be Sick

**Published:** 1878-01

**Authors:** Ben. H. Pratt


// THE BISTOURY.

Vol. XIII. ELMIRA, N. Y., JAN., 1878. No. 4.

### Contributions.

### HOW TO BE SICK.

BEN. H. PRATT.

It is comparatively easy to know how to behave one's self when one is well. It is quite a different thing to know how to behave one's self when one is sick. The most popular people make themselves very disagreeable as soon as something ails them, and no man ever exhibits his idiosyncrasy so surely as when he is physically out of order. A man might save his relatives a great deal of trouble and annoyance by the exercise of a little thoughtfulness and consideration when he isn't very well. It should be every man's aim to make his associates happy, and keep his family from hating him, as far as it lies in his power, and among efforts to do this there is nothing that goes further than exercising a commendable deceitfulness as to one's physical health. Among his acquaintances down town a man should always appear to be in the best possible physical condition. However numerous he knows his maladies are—however demoralized the condition of his stomach—however sluggish the action of his liver—however galloping his brain may be arising from cerebral fever—however painfully his muscles may twinge with every movement, owing to the presence of rheumatism—he should never, by a single tell-tale look, word or action, reveal the disagreeable fact that he is not the personification of absolute physical integrity. This kind of man is welcome everywhere. People may remark upon his bloodshot eyes, or his jaundiced skin, or note him wince a little when his rheumaticky arm is shaken, or remark that his walk is affected by a slightly limpish gait—yet if he only stands up and lies himself out or all this, giving brief excuses for the above phenomena, all will be overlooked in a minute, and nobody will suspect that he's an invalid. Of course he must talk cheerfully, and make jokes upon his misfortune in looking so much like a sick man when he is blessed with such exquisite health; and when he can't stand his pain any longer without mentioning it, he must make a plausible excuse for going to his room, or office, or up some lonely alley, to deliver himself up to his aches and pains and general misery, unseen by mortal eye. The considerate invalid should always have in view the happiness of his fellow men, and never inflict even a hint of his suffering or malady upon them. His heroism should consist in being always well to everybody but himself. A sick man at home, too, is a terrible affliction upon the family, and recognizing this fact, the prudent man will always conceal his indisposition from his wife, children, mother, mother-in-law and old maid sister. If he is taken sick down town he should get home with as little fuss as possible. If he can't catch a car, or, with his face strained to cheerfulness, hail a friend in a buggy and jokingly chaff him into giving him a ride homeward, he should walk or crawl home through all the back alleys he knows of. When within half a block of his own door he should brace up manfully, walk erect, and with his usual alacrity; put on a pleasant expression, and enter his house with a cheery salutation for every inmate, from baby to mother-in-law. If it be mealtime, he must not refuse to eat of everything upon the table, however rich it may be, or however repulsive to his stomach. He should take pains to exhibit an unusual appetite upon such occasions, even though he knows he must endure no end of torture to pay for it. He must enter into the merits of the conversation, and do so with all that ardor and brilliancy which he owes to the good digestion of his household. He should, after the meal is over, either continue the exercise of his entertaining powers, or invent a good excuse for going to bed. The latter he should avoid, however, except under the absolute necessity of doing this or something worse. Once in his room, he may writhe and toss and moan all he chooses, so long as no member of his family is present; in the latter case, however, his instinct of propriety, and his sense of duty, will suggest an absolute concealment of anything like indisposition. If his children insist upon having "a romp with papa" upon the bed, he must submit with a stoicism he owes his offspring, and should his wife conclude to do up her long neglected stitching upon the sewing machine at the head of the bed, no objection to the racket should be raised, lest a hint of illness be conveyed and the home made miserable. It's dreadful to be sick and have your family begin to hate you for interfering with their accusiomed enjoyments. Of course you are not expected to go to sleep, as that would arouse suspicion, so you must smile and say you are only a little tired, or didn't get rest enough the night before, or something like that. But if, by some yielding of your weak nature to an unendurable spasm of pain, you should give vent to a stifled groan, or to an unfortunate expression of the pain that is racking you, and thus let out your secret, don't make a still bigger fool of yourself by carrying your ailments to desperate lengths. The family may think they have discovered that you are really sick now, but it isn't too late to fix matters all up yet, and you can say that you were groaning in spirit over the business depression that prevails. If, however, you have not exercised sufficient tact at this very delicate juncture, you must make the best of it by asserting that you are only a little under the weather, as it were, and beg everybody to go right along with their noise and clatter. Of course your wife will, by this time, have spread a brown paper, saturated with vinegar, on your head, and your mother-in-law will have slapped a square foot of mustard pudding over your stomach, and your sister, in about three minutes, will have a steaming bowl of elder-blow tea ready to drench you internally, all of which you must submit to without a murmur. If you can galvanize your face into a smile it will help matters very materially. This is a very critical point in the struggle of conducting yourself with propriety, and one should carefully retain his presence of mind, for now, however rackingly your head may ache, you will soon be called upon to submit to the rubbing process. Your wife will assure you that there's nothing so good for a headache as rubbing your forehead and temples, and combing your hair with a coarse comb. Resistance will subject you to a charge of ingratitude, and no matter how acutely your temples may ache, and how oppressive a feather's weight thereon would prove, you must endure this rubbing and kneading and pawing process until the muscular energy of your wife is completely exhausted; and then you may indulge a hope of being let alone unless some fresh member of the household is called in to continue the operation. It requires all one's heroism to stand all this, but it's the only way to avoid making your people miserable.

We know men whose homes have been deprived of every enjoyment because of the indiscretion of the head of the household. These men enter their dwellings with the most lugubrious countenances, just because they are sick. Their selfish nature crops out at once. They rub their hands across their foreheads and utter that most doleful of all exclamations, "My gracious! What a headache I've got!" and then the spirits of the family at once fall to zero. There's no fun for anybody after that. The children are hushed up—the playthings are all put away—everybody talks in whispers—anticipations of a pleasant evening are over—the house becomes as gloomy as a cave—and all because one man has a thumping pain in his head. Of course every member of his family begins to hate that man. They don't mean to, but they do. They can't see why the happiness of six or eight well people should be converted into misery because of the sickness of one. And he intensifies this feeling, likely enough, by insisting upon going to bed without any supper. He has so little regard for his family as to decline to load his stomach with what he has no appetite for. He won't have any vinegar and brown paper on his head, and refuses it with a very impatient token of negation. He snubs the mother-in-law who proposes the mustard plaster, and makes her mad. He actually says that the smell of elder-blow tea makes him sicker, if possible, than the smell of onions, and the sister is made an enemy of. He won't submit to have his head rubbed and pawed and combed, and tortured into any more intense agony than that which he already endures. He *will* groan, and he *won't* soak his feet in hot water, and it hurts him to talk, and he wishes they wouldn't ask him any questions or propose any more remedies. He always has to tell them all, once for all, that he has tried all these things, and then wonders why in the name of conscience he can't be let alone, and allowed to remain quiet, and why they conspire together to make him worse. Then he gets up and declares that he'll go out to the barn, or down into the coal bin, or somewhere, and have a little rest. He can't see why a sick man can't be let alone—thinks there's a conspiracy to torture him. Finally he succeeds in driving his folks away from him, and he then begins to enjoy his sickness in his own way, wholly indifferent to the fact that he's made a mortal enemy of every member of the household, and has jeopardized his domestic happiness forever, to a greater or less degree.

Why is it that men, with the experience they all have had, will persist in acting in this very impolite way? They know that nobody enjoys the society of sick people, and therefore one should always conceal his ailments as long as he can, unless he enjoys being avoided. When a man gets so bad that his will power isn't strong enough to conceal his pain, then he should make the best of his circumstances; and while it is of no use whatever to try and make people enjoy his society, he should lend all his energies to keeping folks, and especially his own folks, from entertaining an absolute hatred for him. It is the most natural thing in the world for people to want to do something for a sick person. It is an instinctive trait of almost the entire human family, and nothing will so quickly alienate one's friends as to have their offers of assistance and alleviation spurned. Hence it is a sick man's duty, and one which he owes to society, to accept every offer of service made, every treatment proposed, and to eat and drink every article sent him by his friends when he is sick. It is worthy of close study, this art of being sick, but we regret to say that there are very few men who have attained to any very marked degree of superiority in this art. Men are so brutally selfish that they can't make sacrifices for the people they meet, the people they associate with, and particularly the people they live with. They have got to tell everybody about it if they have a headache, or a toothache, or are colicky, or have corns. They screw up their faces to an agonized wrinklidity, and thus hang out their sign. Then they always have things so much worse than anybody else ever had them before, and have to tell when and where the trouble began, and it's a doleful family history, like enough running through three or four generations. But this wouldn't be so bad if the man only behaved himself at home. Men can have business somewhere, and flee to the uttermost parts of the town to escape a loquacious ailer, but one's family can't escape a sick man, and he ought to consider them in their helplessness, and let his lancinating pain, like the worm i' the bud, feed on his corporality and double himself into a hard knot, before he intimates his condition. When he gives way through mental weakness, then his duty is still plain. He should allow his entire head to be decorated with vinegared paper; he should submit gracefully to have his chest and abdomen spread all over with mustard plasters; he should rejoice that his feet are ample enough to accommodate a fair crop of horse radish leaves; he should feel grateful that his stomach's capacity is equal to any bowl in his pantry, and that he cannot be flooded out by any reasonable amount of those fragrant decoctions of elder-blow, catnip pennyroyal or boneset; he shauld be thankful that his vitality is great, and that no amount of rubbing, and kneading, and pawing, and scalp-scraping can obliterate his existence; he should be mentally thankful that his wife is not a muscular woman, however, and he should whistle—though not audibly—pæans of praise to that effect. If there be any room upon his face unoccupied by the acidified decorations before referred to, he should somehow wiggle in a smile upon the largest bare spot there is, and thus evince his gratitude for the attentions he is receiving at the hands, plasters, papers, leaves and herbs of his devoted family. It may be regarded as somewhat difficult to do this, but compared with the consequences which will result from not doing thus, these are trifling sacrifices. We have never known men to die of vinegar paper, mustard plasters, horse radish leaves or excessive rubbing and combing, and however near unto death a man may deem himself under these manipulations, let him remember that he is safe—at least until a doctor is sent for. He will rise again. Let him mentally ejaculate "resurgam!" for his continued encouragement. It will do him good and enable him to bear up—or down—in hopes of a return to his normal condition in the near future. And when he gets over it, he will have the double satisfaction of knowing that he is a hero, and that he still maintains the position of a friend in his own family, and not an enemy. His baby will not cry when he approaches. His older children will not run and hide under the bed when he enters the house, but at once rush at him, feel in his pockets for candy or nuts, and cry magnificently when they find not the toothsome morsels. His mother-in-law will increase the frequency and length of her visits to his house, and make it pleasant for him by assisting his wife in her arguments upon the necessity of refurnishing the parlor and purchasing a new piano. His wife will throw off that sullen reserve which characterizes many wretched women, and have no hesitancy in asking for a new bonnet four times a year, or presenting her purse weekly for refillment. She will exercise a delicious freedom in requesting him to stop at sundry places on his way to business, and order the most tempting delicacies for the table, and commission him to bring home with him a glorious armful of family wants which have already been ordered. Indeed, his life will be one continued round of blessed domesticity, and he will have his mind so fully occupied thereby that the calls of creditors will cease to annoy him; and when he fails, and experiences the delights of that bankruptcy towards which his sacrificing disposition has led him, he will descend the beautiful paths of much lauded poverty with a light heart and a beaming smile, and within whatever sacrificial depths of destitution he may be called upon to flounder, when he dies the newspapers will close his obituary with that very original and touching phrase, "he was a kind husband, an indulgent father, a sympathizing friend, and leaves a large circle of acquaintances to mourn his untimely decease; also a large family in destitute circumstances, to whose condition the attention of the benevolently inclined is respectfully directed."

